# EndoDB: a database of endothelial cell transcriptomics data

**DOI:** 10.1093/nar/gky997

**Published:** 2018-10-24

**Authors:** Shawez Khan, Federico Taverna, Katerina Rohlenova, Lucas Treps, Vincent Geldhof, Laura de Rooij, Liliana Sokol, Andreas Pircher, Lena-Christin Conradi, Joanna Kalucka, Luc Schoonjans, Guy Eelen, Mieke Dewerchin, Tobias Karakach, Xuri Li, Jermaine Goveia, Peter Carmeliet

**Affiliations:** 1 Department of Oncology and Leuven Cancer Institute (LKI), Laboratory of Angiogenesis and Vascular Metabolism, KU Leuven, 3000 Leuven, Belgium; 2 State Key Laboratory of Ophthalmology, Zhongshan Ophthalmic Center, Sun Yat-Sen University, Guangzhou 510060, Guangdong, P.R. China; 3 Laboratory of Angiogenesis and Vascular Metabolism, Center for Cancer Biology, VIB, 3000 Leuven, Belgium

## Abstract

Endothelial cells (ECs) line blood vessels, regulate homeostatic processes (blood flow, immune cell trafficking), but are also involved in many prevalent diseases. The increasing use of high-throughput technologies such as gene expression microarrays and (single cell) RNA sequencing generated a wealth of data on the molecular basis of EC (dys-)function. Extracting biological insight from these datasets is challenging for scientists who are not proficient in bioinformatics. To facilitate the re-use of publicly available EC transcriptomics data, we developed the endothelial database EndoDB, a web-accessible collection of expert curated, quality assured and pre-analyzed data collected from 360 datasets comprising a total of 4741 bulk and 5847 single cell endothelial transcriptomes from six different organisms. Unlike other added-value databases, EndoDB allows to easily retrieve and explore data of specific studies, determine under which conditions genes and pathways of interest are deregulated and assess reprogramming of metabolism via principal component analysis, differential gene expression analysis, gene set enrichment analysis, heatmaps and metabolic and transcription factor analysis, while single cell data are visualized as gene expression color-coded t-SNE plots. Plots and tables in EndoDB are customizable, downloadable and interactive. EndoDB is freely available at https://vibcancer.be/software-tools/endodb, and will be updated to include new studies.

Endothelial cells (ECs) line the lumen of blood vessels, are metabolically active and orchestrate important processes such as vasomotor tone, coagulation, permeability, tissue vascularization and immune response (1). The inability of ECs to fulfill their physiological functions is a key feature of multiple prevalent diseases including hypertension, atherosclerosis, diabetes and cancer (2). In the past three decades, hypothesis-driven studies have started to unravel the molecular mechanisms that underlie EC function in health and disease, which resulted in clinically approved EC-targeting drugs such as bevacizumab and ranibizumab for cancer and age-related wet macular degeneration, respectively (3). Recent technological advances have made it possible to perform global profiling of transcript (transcriptomics), protein (proteomics) and metabolite levels (metabolomics), even in single cells. These technologies have provided unprecedented insight in EC biology and generated many novel hypotheses (4).

To facilitate the re-use of omics datasets, it is now a standard requirement of most scientific journals to make published data publicly available via repositories such as ArrayExpress (5) and Gene Expression Omnibus (GEO) (6). However, the main purpose of these primary archives is to store raw data in a format that is accessible by bioinformaticians, not to enable bench scientists and experimentalists to directly explore and re-use data to answer biological questions (7). In the cancer field, added-value databases (databases that are specifically designed to allow bench scientists to explore pre-selected, highly curated and pre-analyzed data) have been transformative and facilitated several breakthrough discoveries (8–13). A few independent research groups have made the results of their EC profiling efforts web-accessible (14,15), but currently no comprehensive EC-specific added-value databases exist to facilitate the reuse of gene expression data by the vascular biology community. Here, we describe the development of the endothelial cell database (EndoDB), the first freely available added-value database of EC (single cell) transcriptomics studies, designed to allow vascular biologists and other bench scientists to unlock the untapped potential of publicly available data via an easy-to-use interactive web interface (https://vibcancer.be/software-tools/endodb). The user-interface and functionality of the EndoDB were developed in close collaboration between software developers, bioinformaticians and vascular biologists. The EndoDB is easy-to-use, allows to interactively explore data using powerful statistical and bioinformatics approaches, and has been field-tested and used in several recent publications (16,17).

## MATERIALS AND METHODS

### Retrieval of EC transcriptomics data and database content

**Figure 1.**
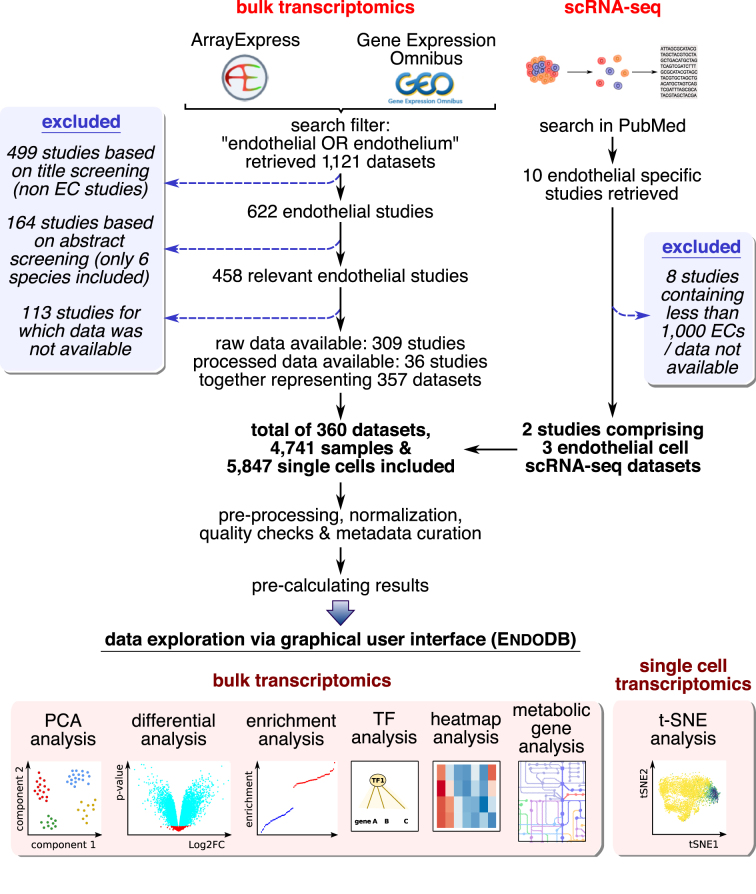
Schematic overview of EndoDB construction and functionalities.

We first aimed to collect and organize EC transcriptomics datasets available in the public domain. To do this, we used a two-step approach (Figure 1 and Supplementary Table S1). First, we used a broad and sensitive filter (‘endothelial OR endothelium’) to search ArrayExpress and GEO for EC transcriptomics datasets, which returned 1121 studies (as of 1 June 2018). We then screened the title, abstract and if necessary sample information to determine which of these studies performed gene expression profiling in ECs specifically. We excluded 663 studies based on the title abstract screen and 113 studies for which data was not made available in the public domain, resulting in a total of 345 studies comprising 357 bulk transcriptomics datasets (a single study can contain multiple independent datasets).

Second, we complemented our search with a PubMed screen to identify recently published single cell transcriptomics studies of ECs, which retrieved 10 single cell RNA sequencing (scRNA-seq) studies (as of 1 June 2018). To ensure high quality scRNA-seq data in the EndoDB, we only considered studies that aimed to perform scRNA-seq in ECs specifically, used isolation protocols optimized for this purpose and sequenced >1000 ECs because: (i) ECs are a minority cell type in all tissues, ranging from ∼1–5% in tumor tissue to ∼20% in highly vascularized organs such as the lung (18,19); (ii) generic dissociation protocols fail to adequately capture ECs, resulting in a further depletion of the EC fraction (20); (iii) ECs are sensitive to dissociation-induced artifacts (20), which biases data interpretation (21), together indicating that dissociation protocols have to be specifically optimized to isolate a high number of high quality ECs; (iv) the reliability of single cell analyses depends on the number of cells analyzed (22,23); and (v) the number of ECs that can be sequenced with more advanced technology (e.g. droplet-based sequencing) will increase by orders of magnitude. Based on these criteria, we excluded eight studies (Supplementary Table S2), and at last included two studies comprising three datasets.

The final database contains 4741 bulk and 5847 single cell EC transcriptomes from six different model organisms (*Homo sapiens* [human], *Mus musculus* [mouse], *Rattus norvegicus* [rat], *Bos taurus* [cow], *Danio rerio* [zebrafish], *Sus scrofa* [pig]), generated using a variety of technologies (micro-arrays, (single cell) RNA sequencing) and platforms (Affymetrix, Agilent, Illumina).

### Pre-processing and quality control of bulk transcriptomics data

To maximize the number of datasets included in the EndoDB, we aimed to include data from all micro-array and RNA-sequencing platforms. For micro-arrays, we retrieved raw data files when possible, but included log2-transformed pre-processed data when raw data was not available. Raw data from the Affymetrix platform was downloaded as binary or text CEL files, normalized using the robust multi-array average using the R-packages *affy* and *oligo*, followed by quantile normalization, log2 transformation and probe-set summarization (24,25). Raw data files from Illumina BeadChips were normalized using the *normexp* algorithm using control probes (neqc) from the *limma* package (26). Raw data from Agilent platforms was normalized using the *normalizeWithinArrays* and *normalizebetweenArrays* functions available from *limma* package (26). Probe set identifiers were mapped to Entrez identifiers, official gene symbols and gene names using annotation packages for the corresponding platform. We applied standard quality controls using the *arrayQualityMetrics* R package to identify low-quality arrays (27). In this approach, samples were scored on the basis of three metrics, the distances between the arrays, Bland–Altman plots (MA plots) and boxplots. Samples that were flagged as outliers based on more than one of these metrics were removed from the analysis in the EndoDB, but the raw data of all samples can be downloaded for downstream analyses in independent software pipelines. At last, we randomly selected 25% of studies and cross-checked our results for congruency with the original publication.

For RNA-sequencing, data were retrieved in fastq format, and alignment to the reference genome and quantification of transcript abundances was performed using the *Kallisto* software (28). We performed trimmed mean of M-values (TMM)-normalization using the R-package *EdgeR* (29) and VOOM-normalization using *limma* before downstream differential analysis.

### Pre-processing and quality control of single cell data

Recent technological breakthroughs have made it possible to RNA sequence transcriptomes of single cells (scRNA-seq) (30). These studies have great resource value and are beginning to elucidate EC heterogeneity at the single cell level. However, so far, no general searchable database of EC-specific scRNA-seq data exists, which limits the broad use of these large-scale sequencing efforts. To increase the resource value of scRNA-seq EC datasets, we have developed a dedicated scRNA-seq EndoDB module. For each dataset, we removed low quality and apoptotic cells based on the number of expressed genes (<200) and the percentage of reads assigned to mitochondrial genes (>5%) (31), and performed library size normalization followed by natural log transformation using log1p available via the *Seurat* package (32). To *in silico* select the ECs in a dataset, we used an unbiased clustering approach, in which we clustered the cells together based on their expression profile and then assessed which clusters express canonical EC marker genes (*PECAM1, CDH5*). We further excluded clusters that expressed markers for leukocytes (*PTPRC*), pericytes (*PDGFRB*) and fibroblasts (*COL1A1*). Using a clustering approach rather than marker gene gating on a cell per cell basis is more robust, since it allows to overcome misclassification of cells as non-EC due to gene drop out.

### Metadata curation

**Figure 2.**
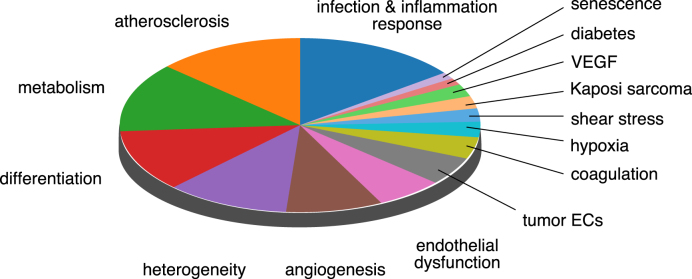
Most common experimental conditions in the EndoDB. Relative representation of the 15 most common sample types in the EndoDB grouped by experimental conditions. Abbreviations—EC: endothelial cell; VEGF: vascular endothelial growth factor.

Metadata was downloaded from GEO or ArrayExpress and manually curated by a team of expert vascular biologists. Repetitive and redundant information was removed, technical information such as the chip design and library preparation was standardized, and each array was manually annotated with biological information such as the model organism, vascular bed, EC type and the experimental conditions (Tables 1–3, Supplementary Table S4 and 5 and Figure 2). In a second step, we used the *OpenRefine* software (33) to standardize spelling across all arrays and datasets. In addition, a description of each study, the GEO or ArrayExpress identifiers and references to the original publication were included in the metadata.

**Table 1.** List of species in the EndoDB

Species	Colloquial species name	Number of datasets in EndoDB
*Homo sapiens*	Human	270
*Mus musculus*	Mouse	76
*Rattus norvegicus*	Rat	5
*Bos taurus*	Cow	3
*Danio rerio*	Zebrafish	3
*Sus scrofa*	Pig	3

Complete list of species included in the EndoDB, the number of datasets per species is indicated.

**Table 2.** Top 10 most common tissue types in the EndoDB

Organ	Number of datasets in EndoDB
Umbilical cord	149
Skin	42
Aorta	28
Lung	28
Heart	25
Brain	22
Liver	16
Eye	15
Blood	13
Lymphatic system	13

Top 10 most common tissue types in the EndoDB, the number of datasets per tissue type is indicated.

**Table 3.** Top 10 most common EC types in the EndoDB

Cell type	Number of datasets in EndoDB
Umbilical vein ECs	142
Aorta ECs	29
Dermal ECs	23
Lymphatic ECs	21
Coronary artery ECs	17
Pulmonary ECs	16
Brain ECs	11
Retinal ECs	11
Dermal lymphatic ECs	10
Blood outgrowth ECs	9

Top 10 most common EC types in the EndoDB, the number of datasets per cell type is indicated.

### Data analysis and visualization

Data visualization greatly facilitates interpretation. For all bulk transcriptomics datasets independently, we performed: (i) principal component analysis (PCA) and visualized the results as 2D plots where individual samples are color-coded according to the experimental design; (ii) differential gene expression analysis (DGEA) (34) and visualized the results as volcano plots, bar plots and browsable tables; (iii) gene set enrichment analysis (GSEA) (35,36) and visualized the results as waterfall plots; and (iv) heatmap analysis to show gene expression changes of a panel of user-selected genes. Together, these analyses cover some of the most commonly used analyses and allow for detailed data interpretation. EC single cell data are provided as interactive t-SNE plots using the *Rtsne* and *plotly* packages (see Supplementary Table S3 for scRNA-seq pre-processing and visualization parameters). All analytical results are pre-calculated and stored in the EndoDB to reduce computation time and to allow fast data retrieval.

### Website implementation

The EndoDB is implemented as an interactive web application using the R/Shiny web framework (37,38). We used the *plotly* package for data visualization and the *data.table* and *DT* packages to provide searchable tables (39).

### Data availability and downloads

All pre-processed data and curated metadata are available for download in comma-separated flat file format. Visualizations can be downloaded as PNG or HTML files that preserve the same interactivity as available from the web application, tables can be downloaded in comma-separated value format.

### Documentation and manual

A detailed user manual and video tutorials are available via the web application.

## EndoDB FUNCTIONALITY

The functionality of the EndoDB is centered on four analytical approaches to extract biologically meaningful information from (single cell) transcriptomics data. Below, we briefly discuss each approach and provide illustrative examples focused on tumor ECs (TECs). TECs are targets of clinically approved anti-angiogenic therapy but remain poorly characterized. Isolating TECs from human or murine tumors is notoriously challenging and laborious, re-use of publicly available data to investigate gene expression reprogramming in TECs is therefore a potentially favorable and cost-effective complementary approach. The examples below are for illustrative purposes only, detailed transcriptome analysis and biological interpretation is beyond the scope of this manuscript.

### Study-centered data exploration

An important purpose of the EndoDB is to facilitate the re-use of publicly available datasets to address novel and unresolved questions, and to allow comparative analyses of similar studies to resolve study-specific biases. The study-centered data exploration options allow users to retrieve studies based on the study title, PubMed identifier, GEO and ArrayExpress identifiers, and manually annotated keywords. After selecting a study, users can download all data and metadata or explore the data using the results from PCA, DGEA, GSEA, heatmap analysis and bar plots.

**Figure 3.**
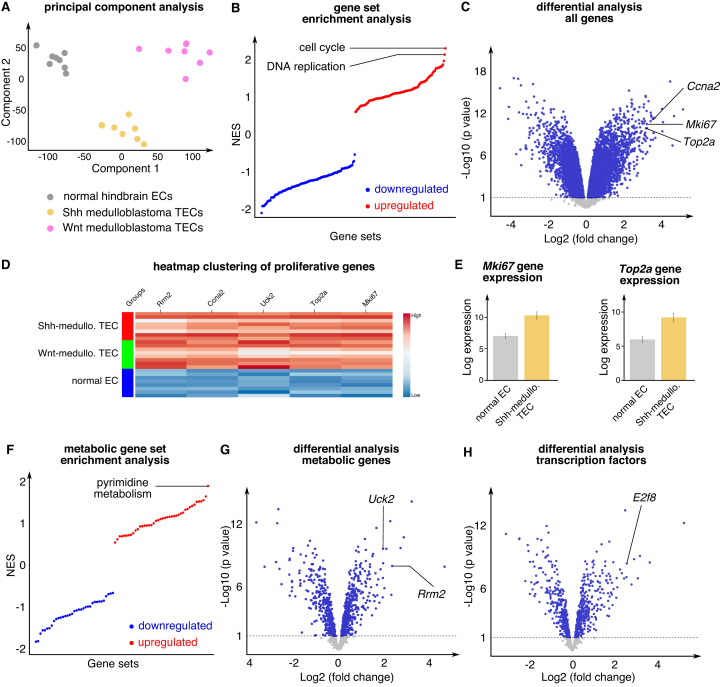
Study-centered data exploration. (**A**) PCA of normal hindbrain, Shh-medulloblastoma and Wnt-medulloblastoma ECs (study E-GEOD-73753). (**B**) Gene set enrichment analysis of normal hindbrain ECs versus Shh-medulloblastoma ECs. The upregulated gene sets are shown in red, the downregulated gene sets are shown in blue. (**C**) Differential analysis of normal hindbrain ECs versus Shh-medulloblastoma ECs shown in volcano plot; some highly deregulated proliferation-associated genes are indicated. (**D**) Gene expression heatmap of normal hindbrain ECs *versus* Shh-medulloblastoma and Wnt-medulloblastoma. The high-gene expression levels are shown in red, the low-expression levels in blue. (**E**) Expression of the indicated genes in normal hindbrain ECs and Shh-medulloblastoma ECs. (**F**) Metabolic gene set enrichment analysis of normal hindbrain ECs versus Shh-medulloblastoma ECs. The upregulated gene sets are shown in red, the downregulated gene sets are shown in blue. (**G** and **H**) Differential analysis of normal hindbrain ECs versus Shh-medulloblastoma ECs for the subset of metabolic genes (G) and transcription factors (H) shown as a volcano plot. Abbreviations—*Ccna2*: cyclin A2*; Mki67*: marker of proliferation ki67; NES: normalized enrichment score; *Rrm2*: ribonucleotide reductase regulatory subunit M2; Shh: Sonic Hedgehog; TEC: tumor endothelial cell; *Top2a*: topoisomerase 2 alpha; *Uck2*: uridine-cytidine kinase 2.

**Example**: We used the study-centered data exploration functionality to re-analyze a previously published dataset on ECs isolated from normal murine hindbrain, Sonic hedgehog (Shh)-driven or Wnt-driven medulloblastoma (40). PCA showed that normal brain ECs (NECs) have a clearly distinct transcriptional profile compared to Shh- and Wnt-driven medulloblastoma TECs (Figure 3A). To determine which pathways are most upregulated in TECs, we assessed differences in gene expression signatures in NECs *versus* Shh-medulloblastoma TECs (Shh-TECs). This analysis revealed upregulation of cell cycle and DNA replication (Figure 3B). Consistently, DGEA showed that several proliferation markers (e.g. *Mki67, Top2a*) ranked in the top 25 most upregulated genes (Figure 3C–E). Together, these data suggest that Shh-TECs undergo an angiogenic switch and adopt a proliferating phenotype.

### Metabolism- and transcription factor-centered data exploration

Emerging evidence indicates that EC metabolism can overrule fundamental signaling cascades, and several metabolic enzymes and transporters have been identified as therapeutic targets to inhibit pathological angiogenesis in ocular disease, inflammation and cancer (41–43). While the importance of EC metabolism is becoming increasingly clear, still little is known about how metabolism supports EC functions in health and disease (44). Analyses of the subset of metabolic genes and gene sets can provide important insight in metabolic reprograming (45,46). We therefore performed metabolic gene expression analysis and metabolic GSEA for all studies in the EndoDB. Similarly, to determine which transcription factors are deregulated in response to experimental manipulation, we performed analysis in the subset of genes encoding transcription factors.

**Example**: Performing metabolic GSEA in Shh-TECs *versus* NECs revealed that pyrimidine metabolism is the most upregulated metabolic pathway. Consistently, metabolic GSEA and DGEA revealed upregulation of several pathways (pyrimidine metabolism) and genes (*Rrm2, Uck2*) involved in nucleotide biosynthesis (Figure 3F and G). Together, these results may indicate that proliferating Shh-TECs upregulate metabolic pathways to sustain nucleotide production required for DNA synthesis. Consistently, the cell cycle related transcription factor *E2f8* was among the most upregulated transcription factors (Figure 3H).

### Gene and pathway-centered data exploration

The EndoDB contains the results of pair-wise DGEA and GSEA results between all experimental conditions within all datasets. The gene- and pathway-centered data exploration functionality allows to unbiasedly determine under which (patho)-physiological and experimental conditions the expression of a particular gene or pathway of interest is most deregulated.

**Figure 4.**
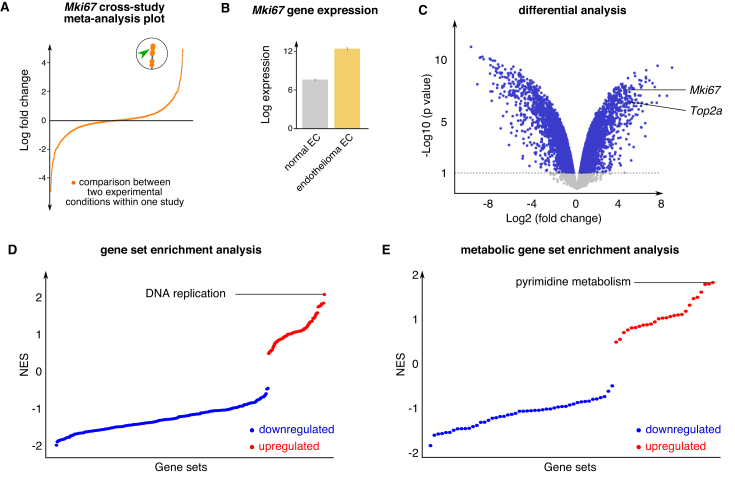
Gene and pathway-centered data exploration, example of *Mki67* gene search. (**A**) Dot plot showing log fold change of *Mki67* gene expression in all sample comparisons in EndoDB datasets. The dot indicated by the green arrow head in the enlarged circle represents the pair-wise comparison selected for further analysis (data from study E-GEOD-14375). (**B**) Expression of the *Mki67* gene in normal hindbrain ECs and endothelioma ECs. (**C**) Differential analysis of normal brain ECs versus endothelioma ECs for all genes shown in volcano plot. *Mki67* and *Top2a* genes are indicated. (**D** and **E**) Gene set enrichment analysis of normal brain ECs versus endothelioma ECs for all genes (D) and for the subset of detected metabolic genes (E). The upregulated gene sets are shown in red, the downregulated gene sets are shown in blue. Abbreviations—*Mki67*: marker of proliferation ki67; NES: normalized enrichment score; *Top2a*: topoisomerase 2 alpha.

**Examples**: Having identified *Mki67* as a gene of interest in Shh-TECs, we interrogated the EndoDB to determine under which other conditions this gene is upregulated. To do this, we performed a gene-centered search, which showed that *Mki67* is highly upregulated in the brain endothelioma cell line (b.End5) compared with primary cultures of normal brain ECs (Figure 4A and B). DGEA and GSEA analysis further showed that *Mki67, Uck2* and DNA replication and pyrimidine metabolism are among the most upregulated genes and pathways in b.End5 endothelioma cells (Figure 4C–E). Together, these findings show that proliferation is a general phenotype of both TECs and malignant ECs.

### Single cell data exploration

An increasing number of studies investigates EC heterogeneity at the single cell level (14,47). scRNA-seq studies are expensive large-scale sequencing efforts that generate high-dimensional datasets with high resource value. We included a specific module in the EndoDB to enable exploration of transcriptional heterogeneity in scRNA-seq datasets via t-SNE plots color-coded for the expression of all detected genes.

**Figure 5.**
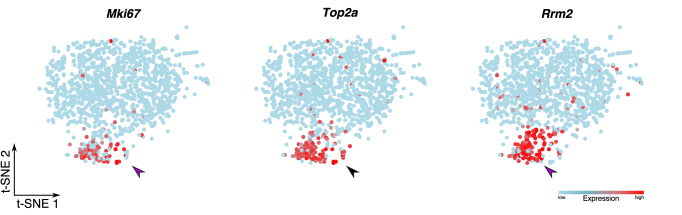
Single cell data exploration. t-SNE plots of COLO205 TECs color-coded for the expression of the indicated proliferation-related genes (GSE110501). Abbreviations—*Mki67*: marker of proliferation ki67; *Rrm2*: ribonucleotide reductase regulatory subunit M2; *Top2a*: topoisomerase 2 alpha.

**Example:** Based on the study- and gene-centered analyses described above, we hypothesized that ECs in tumors adopt a proliferative phenotype. From bulk transcriptomics analysis alone, it is not possible to determine whether all TECs, or only a subset, are actively proliferating. To determine whether the TEC-associated genes *Mki67, Top2a and Rrm2* are upregulated in a specific subset of TECs, we interrogated scRNA-seq data of TECs isolated from a colorectal cancer (COLO205) xenograft model (47). Interestingly, all three genes marked a specific subset of TECs suggesting that the observed upregulation of cell cycle-related genes in bulk transcriptomics data results from increased expression in a specific subset of TECs (Figure 5).

Together, these illustrative examples demonstrate the benefit and power of the EndoDB to capitalize on publicly available data to generate novel testable hypotheses in clinically relevant settings.

## DISCUSSION

EndoDB facilitates bench scientists to unlock the untapped potential of publicly available transcriptomics data but can also be used by bioinformaticians as a resource of expert curated data. EndoDB has been constructed using field-tested methods, is tailored to extract biologically meaningful insight from gene expression profiling efforts, covers emerging fields such as EC metabolism, and is a first-in-class resource of EC scRNA-seq data. EndoDB distinguishes itself from other databases by its flexible user interface, providing an exceptionally large variety of pre-calculated data and analyses in customizable plots and downloadable tables, allowing direct generation and implementation of working hypotheses.

The EndoDB was developed anticipating that an increasing number of EC profiling datasets will become available in the near future. The database can be further expanded by including datasets derived from other vascular cells, such as vascular smooth muscle cells and pericytes, but also to include other omics data such as metabolomics and proteomics.

Since ECs have key roles in health and disease, and (in)directly influence all cells in the body, we expect that EndoDB will be a useful tool, for both vascular biologists and the broader biomedical research community, to derive unprecedented insight in EC heterogeneity, identify new (metabolic) therapeutic targets and generate novel hypotheses. EndoDB is freely available at https://vibcancer.be/software-tools/endodb, and will be regularly updated.

## DATA AVAILABILITY

All pre-processed data and curated metadata are available for download in comma-separated flat file format.

## Supplementary Material

Supplementary Data
