# Supplementary material for: EndoDB: a database of endothelial cell transcriptomics data
Source: Nucleic Acids Res. 2018 Oct 24;47(Database issue):D736–44. doi: 10.1093/nar/gky997 (PMC6324065; doi:10.1093/nar/gky997)
Supplement: Supplementary Data [file gky997_supplemental_files.zip › Carmeliet_EndoDB_supplementary_legends.docx]

**Supplementary Tables**

**Supplementary Table 1:** Data mining steps for inclusion of datasets in EndoDB.

Overview of all datasets evaluated for inclusing in the EndoDB.

**Supplementary Table 2:** Evaluated single cell RNA-seq datasets.

List of all EC-focused single cell RNA-seq datasets evaluated for inclusion in the EndoDB. Datasets including minimum number of ECs (>1,000) were included in the EndoDB (marked in red).

**Supplementary Table 3:** Complete list of datasets included in the EndoDB grouped by cell type.

**Supplementary Table 4:** Complete list of datasets included in the EndoDB grouped by organ type.

**Supplementary Table 5:** Parameters for single cell RNA-seq re-analysis.

List of parameters used for re-analysis of publicly available scRNA-seq datasets including parameters for dimensionality reduction.
